# Do the tuberculin skin test and the QuantiFERON-TB Gold in-tube test agree in detecting latent tuberculosis among high-risk contacts? A systematic review and meta-analysis

**DOI:** 10.4178/epih/e2015043

**Published:** 2015-10-03

**Authors:** Erfan Ayubi, Amin Doosti-Irani, Ehsan Mostafavi

**Affiliations:** 1 Department of Epidemiology, Pasteur Institute of Iran, Tehran, Iran; 2 Department of Epidemiology and Biostatistics, School of Public Health, Tehran University of Medical Sciences, Tehran, Iran; 3 Research Center for Emerging and Reemerging Infectious Diseases, Pasteur institute of Iran, Akanlu, Kabudar Ahang, Hamadan, Iran

**Keywords:** Kappa, Meta-analysis, Latent tuberculosis

## Abstract

**OBJECTIVES:**

The QuantiFERON-TB Gold in-tube test (QFT-GIT) and the tuberculin skin test (TST) are used to diagnose latent tuberculosis infection (LTBI). However, conclusive evidence regarding the agreement of these two tests among high risk contacts is lacking. This systematic review and meta-analysis aimed to estimate the agreement between the TST and the QFT-GIT using kappa statistics.

**METHODS:**

According to the Preferred Reporting Items for Systematic Review and Meta-Analyses guidelines, scientific databases including PubMed, Scopus, and Ovid were searched using a targeted search strategy to identify relevant studies published as of June 2015. Two researchers reviewed the eligibility of studies and extracted data from them. The pooled kappa estimate was determined using a random effect model. Subgroup analysis, Egger’s test and sensitivity analysis were also performed.

**RESULTS:**

A total of 6,744 articles were retrieved in the initial search, of which 24 studies had data suitable for meta-analysis. The pooled kappa coefficient and prevalence-adjusted bias-adjusted kappa were 0.40 (95% confidence interval [CI], 0.34 to 0.45) and 0.45 (95% CI, 0.38 to 0.49), respectively. The results of the subgroup analysis found that age group, quality of the study, location, and the TST cutoff point affected heterogeneity for the kappa estimate. No publication bias was found (Begg’s test, *p*=0.53; Egger’s test, *p*=0.32).

**CONCLUSIONS:**

The agreement between the QFT-GIT and the TST in diagnosing LTBI among high-risk contacts was found to range from fair to moderate.

## INTRODUCTION

According to the most recent statistics, nine million people were infected with tuberculosis (TB) in 2013 worldwide, and 1.5 million died from the disease. It is estimated that 37 million lives have been saved through the diagnosis and treatment of TB between 2000 and 2013 [1]. Close contacts with patients with sputum smear-positive and culture-confirmed *Mycobacterium tuberculosis* have been shown to be at a higher risk for developing latent TB infections (LTBIs), which can be followed by overt TB disease [2,3]. An effective way to disrupt the transmission of infection and to improve disease control is tracing the contacts of TB patients, as well as diagnosing and performing interventions against LTBIs [4,5].

The tuberculosis skin test (TST), also known as the tuberculin test or the purified protein derivative (PPD) test, has been widely used to determine if a patient shows an immune response to the bacterium that causes TB. The TST has therefore been used as a screening tool to detect LTBI in developed and developing countries. However, some inherent difficulties exist in interpreting TST results; false positives may occur due to cross-reactivity with antigens against non-tuberculous mycobacteria and in patients who have received the Bacillus Calmette-Guérin (BCG) vaccine against tuberculosis [6]. In addition, in individuals with a weakened immune system due to conditions such as HIV, false negative results have been observed [7]. In order to address the challenges posed by the TST, the QuantiFERON^®^-TB Gold in-tube test (QFT-GIT) and the T-SPOT^®^ TB test (T-Spot) have been introduced as new diagnostic tests for LTBI. QFT-GIT is a qualitative laboratory test using whole blood specimens to assess the presence of LTBI [8,9]. Studies have shown that the QFT-GIT assay has a comparable sensitivity to the TST, as well as superior specificity, negative predictive value, and positive predictive value [10-12]. Many studies have investigated the level of agreement between the TST and the QFT-GIT in close contacts of patients with active pulmonary TB. They found that the agreement of two test can be vary from poor to moderate [13-15].

The agreement of the TST and the QFT-GIT in detecting LTBI in recent contacts of infectious sources (e.g., index cases) has so far only been addressed in several individual studies. It has been found that the range of agreement, as shown by the kappa coefficient, among studies in different regions in the world is inconsistent, due to heterogeneity in variables such as age, country, and BCG vaccination. For example, in two different studies in pediatric and adult contacts, the kappa coefficients were 0.52 and 0.07, respectively [13,16]. Obtaining a unified estimate by pooling individual studies may play a fundamental role in determining which test is more accurate, whether these two tests are interchangeable, and identifying the proper procedure for diagnosing LTBI in different contexts. Thus, the aim of the present meta-analysis was to estimate the overall agreement, as shown by the kappa coefficient, between the TST and the QFT-GIT in individuals who had been in contact with cases of sputum smear-positive and/or confirmed-culture TB.

## MATERIALS AND METHODS

### Search strategy and selection criteria

The major international scientific databases, including PubMed, Scopus, and Ovid, were searched for articles published as of June 2015 using the following keywords: latent tuberculosis infection, QuantiFERON, interferon-gamma release test, interferon-gamma release assay, enzyme-linked immunospot assay, tuberculin test, PPD-S, skin test, Mantoux tuberculin skin test, kappa, kappa value, kappa statistic, agreement, observational study, cross-sectional study, cross-sectional analysis, cross sectional survey, cohort study, retrospective study, prospective study, and human. Full-text articles were reviewed when the abstracts did not provide sufficient information to determine whether an article was appropriate for inclusion. Furthermore, the reference lists of the retrieved articles were examined for additional relevant studies. If there were any the missing, incomplete, and unreported variables, email communication with corresponding authors of articles was considered to elucidate the data.

### Inclusion criteria for studies

Studies were included if they reported the LTBI screening of high-risk participants with no TB diagnosis who lived in the same household or neighborhood as active pulmonary TB patients diagnosed by positive acid-fast bacillus smears and/or cultures, and contained original data that could be used to calculate the agreement coefficient (kappa) and the standard error (SE) kappa. In addition, Studies that blood samples were collected before administration of the Mantoux TST test and the QFT-GIT cutoff value as 0.35 IU/mL.

### Data extraction and quality assessment

Two investigators (Erfan Ayubi and Amin Doosti-Irani) independently screened the titles and abstracts of the retrieved citations in order to identify the studies that were relevant. In the next stage, the full texts of the relevant studies were examined in order to determine which studies met the eligibility criteria. Two investigators (Erfan Ayubi, Amin Doosti-Irani) independently reviewed and extracted the data from the studies that were ultimately included. Any disagreements were resolved by the third author (Ehsan Mostafavi). The extracted data included the following variables: first author, publication year, country, sample size, mean or median age, history of BCG vaccination, and TST induration diameter. A modified checklist from the Strengthening the Reporting of Observational Studies in Epidemiology (STROBE) statement was applied to assess the quality and risk of bias in the studies included in the meta-analysis [17]. Based on the STROBE criteria, the following seven items were used to assess the risk of bias and quality: (a) a clear definition of the study population; (b) description of the setting, locations, and relevant dates; (c) an exact definition of the outcome, such as LTBI diagnosis by the TST and/or the QFT-GIT; (d) eligibility criteria for the participants; (e) an explanation of how the study size was determined; (f) figures reflecting the number of outcomes associated with each test; and (g) an explanation of when each test was conducted, such as whether blood sampling for the QFT-GIT took place before the TST. Two authors (Erfan Ayubi, Amin Doosti-Irani) assessed the quality and risk of bias in the studies that were included using the above criteria. Studies that fulfilled all of the above criteria were classified as having a low risk of bias. Studies that met one criteria were classified as having an intermediate risk of bias, and studies fulfilling more than one criteria were classified as having a high risk of bias.

### Statistical methods

A 2×2 contingency table was constructed with the number cases with positive TST and negative QFT-GIT results, the number of negative TST and positive QFT-GIT results, the number of positive TST and positive QFT-GIT results, and the number of negative TST and negative QFT-GIT results. Indeterminate results of the two tests were considered meaningless. The kappa statistic was calculated to assess the level of agreement between the TST and the QFT-GIT in each study. SE and a 95% confidence interval (CI) for kappa were calculated using the methods described by Fleiss et al. [18]. Judgments of the kappa estimates were performed according to the criteria articulated by Landis & Koch [19].

In this study, heterogeneity was assessed by I-squared indices [20]. I-squared is the percentage of total variation across studies that is due to heterogeneity rather than chance. I-squared values lie between 0% and 100%. A value of 0% indicates no observed heterogeneity, while larger values show increasing heterogeneity. Following the suggestion of Higgins et al. [20] I-squared values <25%, 25-75% and >75% were considered to indicate low, moderate, and high heterogeneity, respectively. Subgroup analysis was applied to determine which characteristics of studies were responsible for statistical heterogeneity among the results of the studies that were included [21]. Egger’s test was performed to examine potential publication bias [22]. In order to identify the effects of prevalence and bias, prevalence and bias indices were calculated and the kappa statistic was adjusted for low or high prevalence and bias using the prevalence-adjusted bias-adjusted kappa (PABAK) method [23].

The extracted data were analyzed in a random effect model using the inverse variance approach [24]. Data analysis was performed using STATA version 11.0 (Stata Corp., College Station, TX, USA).

## RESULTS

**Figure 1.**
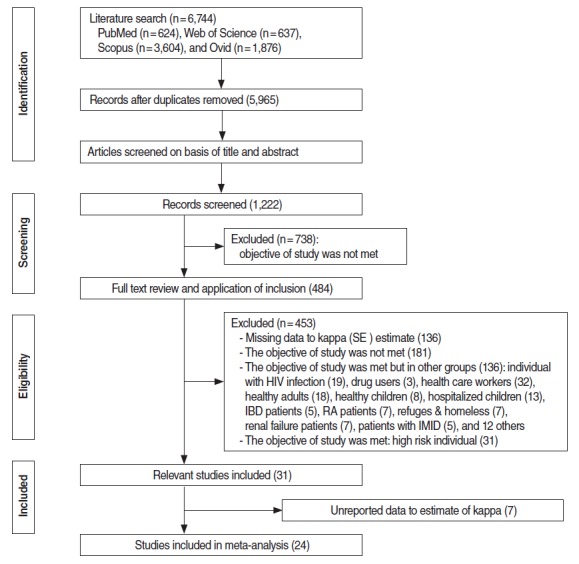
The flow chart of retrieve studies into meta-analysis. SE, standard error; IBD, Inflammatory Bowel Disease; RA, Rheumatoid Arthritis; IMID, Immune-Mediated Inflammatory Diseases.

A total of 6,744 citations were retrieved from the electronic databases. After an initial screening of the titles and abstracts utilizing the abovementioned criteria, 31 articles were identified for detailed full-text review and data extraction. Seven articles were excluded [25-31] due to insufficient and/or unreported data that made it impossible to calculate kappa values, and 24 articles were ultimately included in the meta-analysis (Figure 1) [4,12,13,15,16,32-50]. Of these studies, two were conducted in the Americas [15,44], nine in Europe [4,16,33-37,41,46], seven in Asia [12,38,40,42,43,47,48], and five in Africa [13,32,39,45,49,50]. All studies included subjects of both sexes. The total sample size of the studies included in the meta-analysis was 13,208. Quality assessment of the studies showed seven studies of low quality [13,16,32,37,38,43,45], eight intermediate-quality studies [15,33,35,36,39,41,47,48] and eight high-quality studies (Table 1) [12,16,34,40,42,44,46,49].

**Figure 2.**
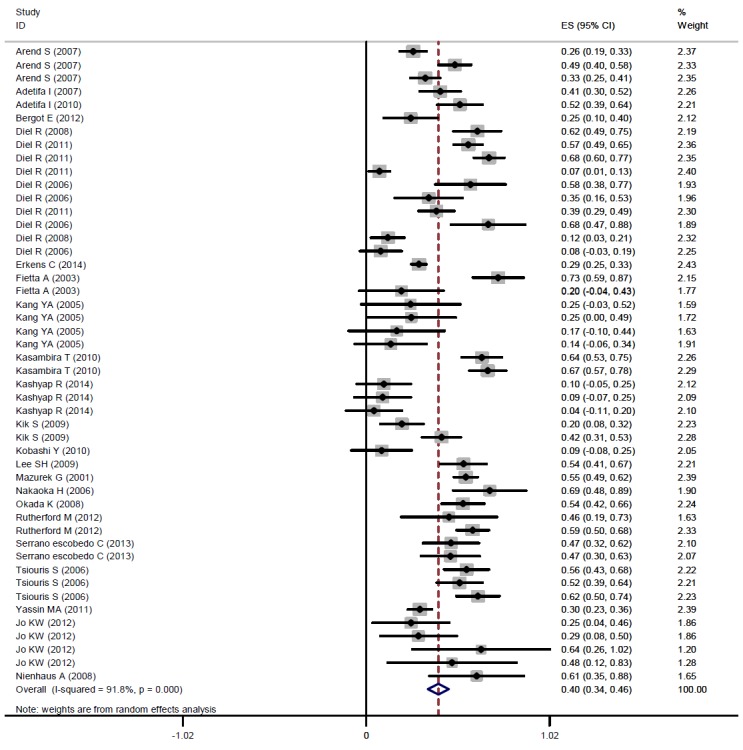
The pooled kappa coefficient for agreement between TST and QFT-GIT among people with high-risk contacts. TST, tuberculin skin test; QFT-GIT, QuantiFERON-TB Gold in-tube test; ES, effect size.

The pooled kappa was 0.40 (95% CI, 0.34 to 0.45) (Figure 2).The results of the subgroup analysis showed that the kappa estimate was statistically significant (p<0.001) according to age group, the quality of the study, location, the burden of TB, and the TST cutoff point. In adults, a pooled kappa of 0.35 (95% CI, 0.28 to 0.41) was found, and in children, moderate agreement was found (0.55; 95% CI, 0.46 to 0.64). Increased values of the cutoff for the induration diameter leading to improved agreement between the two tests. The lowest and highest levels of agreement were observed in Asian and African studies, respectively: 0.29 (95% CI, 0.18 to 0.41) and 0.55 (95% CI, 0.43 to 0.64), respectively (Table 2).

In the sensitivity analyses, the use of PABAK did not materially change the compared kappa estimate. The overall PABAK estimate was 0.45 (95% CI, 0.38 to 0.49), and the PABAK estimates for adults and children were 0.38 (95% CI, 0.28 to 0.49) and 0.60 (95% CI, 0.51 to 0.70), respectively (Table 2).

**Figure 3.**
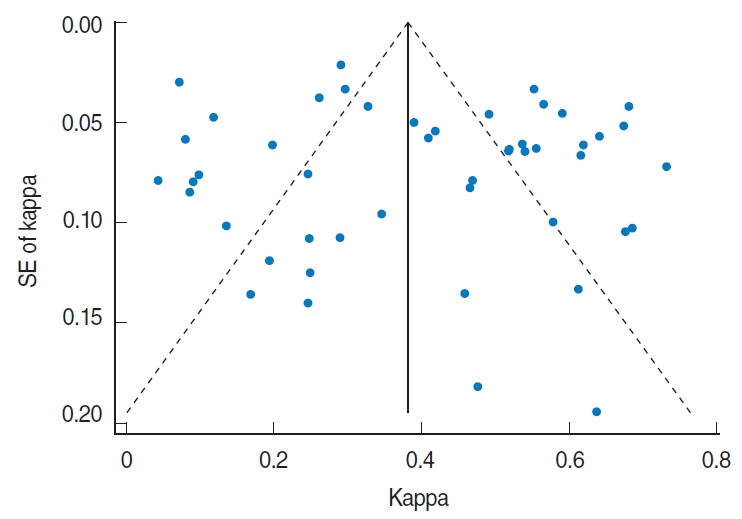
Funnel plot, using data from included studies in meta-analysis, with kappa displayed on the horizontal axis and SE (kappa) on the vertical axis; symmetrical plot shows the absence of publication bias. SE, standard error.

Visual inspection of the funnel plot indicated some asymmetry in the studies included in the meta-analysis (Figure 3). Begg’s test and Egger’s test did not show significant evidence of publication bias (Begg’s test, *p*=0.53; Egger’s test, *p*=0.32).

## DISCUSSION

To the best of our knowledge, this was the first meta-analysis to estimate the agreement between the QFT-GIT and the TST in the detection of LTBI in individuals with high-risk contacts. The results indicate a fair level agreement between the two tests. In studies with no prevalence no bias effects, the kappa estimate showed a moderate level of agreement. Subgroup analysis determined that the agreement between two tests was affected by age group, the quality of the studies, their location, and the TST cutoff point.

This meta-analysis demonstrated fair agreement with regard to heterogeneity among the studies. This fair level of agreement is consistent with other meta-analyses of high-risk individuals, which found kappa values of 0.28 among healthcare workers (95% CI, 0.22 to 0.35) [51].

An important issue that has been explored in some primary studies is the concordance between interferon-gamma release assays and the TST in BCG-vaccinated persons [12,13,46]. In present study, heterogeneous reporting of individual studies and the inability to identify participants who had undergone BCG vaccination in all studies precluded meta-analysis according to BCG vaccination status. Nienhaus et al. [46] found that BCG vaccination was responsible for 81.5% of TST+/QFT− cases; in other words, BCG-vaccinated individuals showed more positive TST reactions, while the accuracy of QFT-GIT was unaffected. This may be explained as a result of false positive TST reactions in individuals with a history of BCG vaccination in developing countries, in contrast with other locations, where the BCG vaccine is often administered at an older age [50]. However, in unvaccinated subjects, these two tests had similar rates of positive results [4].

Another variable that can be considered to have affected our findings is how the degree of contact with the index case was measured. The definition of a contact was not always clear in individual studies. In one study, close contacts were defined as all contacts who had a minimum of 40 hours of exposure to their respective index case [4], while in another study, close contacts were defined as individuals who had household contact in the same rooms with smear-positive pulmonary TB patients for more than eight hours per day [12]. Close contacts with active TB patients can be considered as one factor that leads to positive QFT-GIT test results among TST-positive subjects. Lee et al. [43] argued that a high rate QFT-GIT+/TST+ results occurred among high-risk contacts due to prolonged close contacts with infectious TB patients

It has been determined that discrepancies between the QFT-GIT test and the TST may be due to the inaccuracies of eachtest. The peptides used in the QFT-GIT can be dissimilar to the spectrum of antigenicity of *M. tuberculosis*, and a borderline result of the QFT-GIT test can affect the QFT-GIT result [38,52]. TST results can be influenced by factors such as incorrect administration, the imprecise interpretation of reactions, and interference caused by previous BCG vaccination [6,38].

Our subgroup analysis showed that the use of a conservative cutoff point for positive TST results (≥15 mm) led to an increased level of agreement, which can be explained as the result of fewer false positive TST results. One study showed that the proportion positive TST results according to the TST cutoff point and positive immunoglobulin-gamma release assay results were varied significantly, which they concluded may have been due to false positive TST results and false negative immunoglobulin-gamma release assay results [12].

In concordance with our results, another meta-analysis of healthy adults and children showed a fair level of agreement between the TST and the QFT-GIT (kappa, 0.35; 95% CI, 0.25 to 0.45) (Appendix 1).

Our analysis has strengths and limitations. The primary strength of this study is that this is the first meta-analysis of kappa and prevalence-adjusted bias-adjusted kappa in the ability of the TST and the QFT-GIT to detect LBTI among high-risk contacts. Given the presence of highly notable heterogeneity, our results should be interpreted with caution; however, a high level of heterogeneity for pooled worldwide data can be expected. Potential factors that were not considered in the present meta-analysis, such as BCG vaccination status or TB burden, may have contributed to the variability found among studies.

In summary, fair agreement was found between the TST and the QFT-GIT in detecting LTBI among the contacts of active TB patients, meaning that no clear recommendation can be made regarding which test is more appropriate to use in patients with high-risk contacts. Further meta-analyses dealing with issues such as agreement between the T-SPOT test and the TST, the agreement between the QFT-GIT and the TST in detecting active TB in high-risk contacts, and metrics such as sensitivity, specificity, and positive predictive value are recommended.

## Figures and Tables

**Table 1.** Characteristics of the studies included in the meta-analysis

First author	Publication year	Country	Sample size	Age (yr)	TST cutoff point (mm)	a	b	c	d
Adults									
Diel R	2011	Germany	459	29 (11.8)^1^	5	108	5	87	259
Diel R	2011	Germany	495	29 (11.8)^1^	5	83	2	326	84
Diel R	2011	Germany	459	29 (11.8)^1^	10	75	38	12	334
Diel R	2011	Germany	495	29.02 (11.8)^1^	10	63	22	92	318
Mazurek G	2001	USA	947	39 (18, 87)^2^	10	146	73	79	649
Kobashi Y	2010	Japan	125	41.8 (9.8)^1^	5	34	16	44	31
Kashyap R	2014	India	162		5	71	7	68	16
Kashyap R	2014	India	162		10	34	44	33	51
Kashyap R	2014	India	162		15	19	59	13	71
Jo KW	2012	South Korea	22	39.9 (17.7)^1^	5	15	1	2	4
Jo KW	2012	South Korea	22	39.9 (17.7)^1^	10	10	6	0	6
Jo KW	2012	South Korea	79	39.9 (17.7)^1^	5	29	9	21	20
Jo KW	2012	South Korea	79	39.9 (17.7)^1^	10	24	14	14	27
Serrano Escobedo C	2013	Mexico	123	42 (16.1)^1^	5	42	9	24	48
Serrano Escobedo C	2013	Mexico	123	42 (16.1)^1^	10	31	20	11	61
Diel R	2008	Germany	278	27.7 (12)^1^	5	32	0	155	91
Diel R	2008	Germany	323	27.7 (12)^1^	5	30	4	26	263
Kang YA	2005	South Korea	48	41 (16, 70)^3^	10	17	17	4	10
Kang YA	2005	South Korea	72	28 (25, 36)^3^	10	7	36	0	29
Kang YA	2005	South Korea	48	41 (16, 70)^3^	15	13	10	8	17
Kang YA	2005	South Korea	72	28 (25, 36)^3^	15	7	24	0	41
Adetifa I	2007	Gambia	194	28 (20, 37)^4^	10	69	33	16	57
Fietta A	2003	Italy	66	39 (23, 75)^2^	10	11	23	4	28
Fietta A	2003	Italy	93	39 (23, 75)^2^	10	31	10	2	50
Erkens C	2014	Denmark	1,828		10	538	92	606	592
Bergot E	2012	France	147	44.5 (18)^1^	10	28	7	50	60
Arend S	2007	Netherland	785		5	448	256	1	80
Arend S	2007	Netherland	785		10	518	186	7	74
Arend S	2007	Netherland	785		15	611	93	13	68
Diel R	2006	Germany	157	28.5 (10.5)^1^	5	47	0	96	14
Diel R	2006	Germany	157	28.5 (10.5)^1^	10	107	0	36	14
Diel R	2006	Germany	152	28.5 (10.5)^1^	10	132	6	3	11
Diel R	2006	Germany	152	28.5 (10.5)^1^	5	122	3	13	14
Kik S	2009	Netherland	282		10	142	10	97	33
Kik S	2009	Netherland	282		15	117	35	46	84
Lee SH	2009	South Korea	185	41 (16, 70)^2^	10	97	11	29	48
Nienhaus A	2008	Germany	181	31.6 (12.7)^1^	10	7	3	5	166
Children									
Okada K	2008	Cambodia	217	-	10	28	19	5	143
Rutherford M	2012	Indonesia	299	4.5 (2, 120)^3^	10	121	35	22	114
Rutherford M	2012	Indonesia	72	6 (13, 117)^3^	10	6	9	1	53
Adetifa I	2010	Gambia	215	-	10	43	29	14	127
Tsiouris S	2006	South Africa	184	9 (5, 15)^2^	5	51	10	33	90
Tsiouris S	2006	South Africa	184	9 (5, 15)^2^	10	51	10	29	94
Tsiouris S	2006	South Africa	184	9 (5, 15)^2^	15	49	12	20	103
Kasambira T	2010	South Africa	239	6 (3, 9)^4^	5	56	19	12	149
Kasambira T	2010	South Africa	236	6 (3, 9)^4^	10	48	27	7	154
Nakaoka H	2006	Nigeria	57	7.4 (3.8)^1^	10	34	6	2	15
Yassin MA	2011	Ethiopia	335	8 (1, 15)^3^	10	87	24	39	59

a, subjects with positive QFT-GIT and positive TST results; b, subjects with negative TST and positive QFT-GIT results; c, subjects with positive TST and negative QFT-GIT results; d, subjects with negative TST and negative QFT-GIT results; QFT-GIT, QuantiFERON-TB Gold in-tube test; TST, tuberculin skin test; TB, tuberculosis.

1 Mean (standard deviation).

2 Mean (range).

3 Median (range).

4 Median (interquartile range).

**Table 2.** Subgroup analysis of kappa and PABAK by quality of study and location (continent) using the chi-squared test for heterogeneity

	Kappa^1^ (95% CI)	I^2^ (%)	p-value	PABAK (95% CI)	I^2^ (%)	p-value
Age group						
Adults	0.35 (0.28, 0.41)	91.6	<0.001	0.38 (0.28, 0.49)	82.3	< 0.001
Children	0.55 (0.46, 0.64)	84.7	<0.001	0.60 (0.51,0.70)	75.0	< 0.001
Quality of study						
High	0.31 (0.20, 0.43)	93.8	< 0.001	0.32 (0.15, 0.49)	86.9	< 0.001
Intermediate	0.46 (0.38, 0.54)	91.0	<0.001	0.54 (0.43, 0.65)	81.5	< 0.001
Low	0.42 (0.29, 0.54)	88.8	<0.001	0.43 (0.25, 0.60)	79.3	< 0.001
Location						
Asia	0.29 (0.18, 0.41)	85.7	< 0.001	0.32 (0.19, 0.45)	80.0	< 0.001
Europe	0.35 (0.28, 0.47)	94.0	<0.001	0.42 (0.27, 0.56)	84.5	< 0.001
America	0.53 (0.47, 0.58)	75.4	< 0.001	0.56 (0.40, 0.71)	70.1	< 0.001
Africa	0.55 (0.43, 0.64)	87.5	< 0.001	0.57 (0.45, 0.69)	81.7	< 0.001
TST cut off point (mm)						
≥5	0.35 (0.22, 0.48)	94.7	< 0.001	0.37 (0.11,0.55)	83.4	< 0.001
≥10	0.37 (0.22, 0.52)	89.0	< 0.001	0.43 (0.22, 0.63)	80.3	< 0.001
≥15	0.43 (0.36, 0.49)	91.8	< 0.001	0.48 (0.39, 0.57)	82.6	< 0.001

The p-values test for heterogeneity. TST, tuberculin skin test; PABAK, prevalence-adjusted bias-adjusted kappa.

1 According to a random effect model.

## Appendix Appendix 1.

The pooled kappa coefficient for agreement between TST and QFT-GIT among healthy subjects. TST, tuberculin skin test; QFT-GIT, QuantiFERON-TB gold in-tube test; ES, effect size.
